# Power and Time Slot Allocation in Cognitive Relay Networks Using Particle Swarm Optimization

**DOI:** 10.1155/2013/424162

**Published:** 2013-10-08

**Authors:** Pouya Derakhshan-Barjoei, Gholamreza Dadashzadeh, Farbod Razzazi, S. Mohammad Razavizadeh

**Affiliations:** ^1^Department of Electrical Engineering, Science and Research Branch, Islamic Azad University, P.O. Box 775/14515, Tehran 1477893855, Iran; ^2^Electrical and Electronic Engineering Department, Shahed University, Persian Gulf Highway, Tehran, Iran; ^3^Department of Electrical Engineering, Iran University of Science & Technology, Tehran, Iran

## Abstract

The two main problems in cognitive radio networks are power and time slot allocation problems which require a precise analysis and guarantee the quality of service in both the primary and secondary users. In this paper, these two problems are considered and a method is proposed to solve the resulting optimization problem. Our proposed method provides an improved performance in solving the constrained nonlinear multiobject optimization for the power control and beamforming in order to reach the maximum capacity and proper adaption of time slots, and as a result a new scheme for joint power and time slot allocation in cognitive relay networks is proposed. We adopt space diversity access as the secondary users access scheme and divide the time between multiple secondary users according to their contribution to primary user's transmission. Helping primary users provides more opportunities for secondary users to access the channel since the primary users can release the channel sooner. In contrast, primary network leases portion of channel access time to the secondary users for their transmission using particle swarm optimization (PSO). Numerical studies show good performance of the proposed scheme with a dynamic cost function in a nonstationary environment.

## 1. Introduction

Cognitive radio (CR) is an intelligent radio communication system which has the potential to make the best of unoccupied licensed spectrum while introducing little interference to licensed or primary user (PU). Cognitive radio is aware of the changing radio environment and is to make real-time adaptation to achieve its targets. Due to the accelerated deployment of broad band communication systems, current fixed frequency allocation schemes spectrum is becoming a major bottleneck. Therefore, there is an increasing interest in this technology among the researchers in academia, industry and spectrum policy makers. Hence, many studies featuring recent advances in theory, design, and analysis of cognitive wireless radio networks have been figured out. In general, cognitive wireless radio network is capable of adapting to the outside existing time varying environment. The cognitive transmitters should use the environmental information to approach the appropriate parameters, such as modulation type, modulation index, coding format, and transmission power level, in order to maximize their data transmission rates under a constrained interference level. One of the main concerns of the networks topology and capacity is the transmission power. To avoid the interference with the primary users, the transmission power of the cognitive wireless radio network should be controlled and limited. Controlling of the transmission power is of vital importance. In [1–3] the power control and spectrum sharing limitations have been studied. According to the descriptions in [4] the power control had an effective impact on the probability of bit error rate. Therefore, in our simulations, minimizing the bit error rate is the optimization criterion. In [5], a relay scheme has been used for balancing the traffic requests and available spectrum resources in cognitive radio. We extended that idea to consider the secondary network as a relay network in our proposed TDMA scheme for balancing the traffic. Signal-to-interference-plus-noise ratio (SINR) has been enhanced by relays through spatial diversity in [6, 7]. In addition, in [8], directional transmission of relays for exploiting spatial spectrum holes has been studied. According to [9], the relay channels make improvement in the performance through spatial diversity by using additional paths between source and destination. Joint beamforming and power control using weighted least square algorithm have been performed in [10]. Beamforming can be implemented either at the transmitter or at the receiver. Transmitter beamforming concentrates the transmission signal on a certain direction in order to minimize interference with other users. Receiver beamforming is usually useful for signal localization or for taking advantage of spatial diversity. In addition, as mentioned in [11], the distances between base stations and users have an impressive role in the topology of the systems. The spectrum sensing and signal localization have been studied in [12, 13]. A power control method based on genetic algorithm for cognitive radio has been presented in [14]. The problem of transmission power control in cognitive radio networks considering propagation channels has been studied in [15–17]. One of the different features of our proposed method is its utilization in randomly time varying fading channels. In [18, 19], a cooperative communication network for cognitive radios has been investigated. Also, the population adaptation for genetic algorithm based on cognitive radio and bioinspired algorithm for dynamic resource allocation and parameter adaptation have been studied in [15–20]. A precise power control in a randomly time varying environment has been studied by employing an intelligent algorithm in [21, 22]. In addition, dynamic spectrum sensing and spectrum management have been studied in [23–25]. The analysis of detection time for multiple-user cooperative spectrum sensing and best relay selection has been studied in [23]. These researches make it possible for a secondary or cognitive radio network to opportunistically utilize a frequency band initially allocated to a primary network. In [26–29], common spectrum sensing and adaptive power allocation methods have been considered. However, evolutionary power control for cognitive users has not been previously investigated in randomly time varying fading environments considering relay diversity and time slot allocation. In addition, the use of PSO in wireless scenario always faces the problem of computational complexity. The wireless channel varies quickly, and the cognitive radio network requires high number of operations in short time intervals. In [30], by using PSO, the minimum bit error rate has been investigated for multiuser transmission design and computational complexity has been figured out for MIMO channel.

 In this paper, we proposed an intelligent method to adapt the channel time slots and allocate transmission power of secondary users (SUs) with relay diversity by using particle swarm optimization. To achieve these goals, we formulate an optimization problem, considering a heuristic strategy for both networks, so we adapt space diversity access for the secondary users access scheme and divide the time between multiple cognitive users regarding their contribution to primary user's transmission. The cognitive user who contributes more to a primary user gets more revenue. Multiple antennas have been assumed to be deployed at the secondary users. Many wireless network standards provision the use of transmit antenna arrays. In our proposed scheme, by using beamforming at the remaining time slot, it is possible to allocate energy in the direction of the intended users, whose channels can often be accurately estimated. Beamforming has been also exploited as a strategy that can serve many users at similar throughput. Due to the variation of radio channel characteristics, as well as the frequency spectrum band availability, cognitive radio networks need to support time varying quality of service requirements. The basic goals of our work are focused on dynamic power and time slot allocation in time division multiplexing access mode, maximizing the transmission rate of both primary and secondary users and minimizing the transmission power of secondary users, with relay diversity in a randomly time varying environment. 

## 2. System Model 

**Figure 1:**
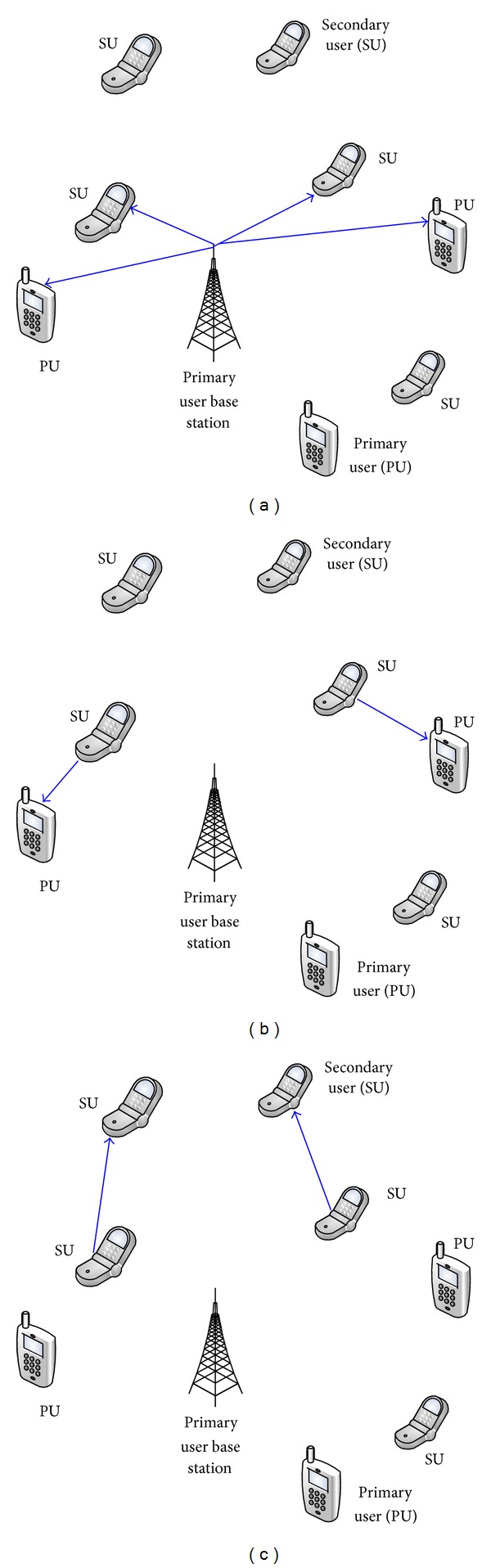
Conceptual diagram of the system model.

**Figure 2:**
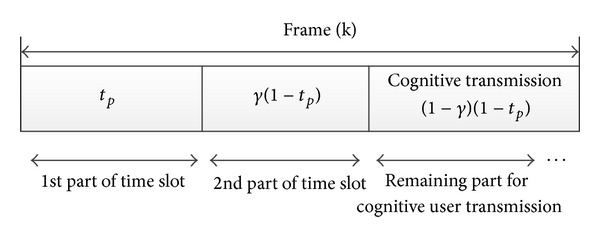
Time slot division for secondary and primary transmission.

We consider a system model where the primary network consists of *N* primary users (PUs) each having a transceiver. The primary network transmits and communicates with the constant and specific transmission power. The system model of our scenarios is illustrated in Figure 1, and the time slot division is shown in Figure 2. The secondary network included *M* secondary users communicating in an ad hoc scenario. The secondary network processing is based on beamforming at both the transmitter (*K* antennas) and the receiver (*K* antennas) for each secondary user link. In our scenario, the downlink of the primary network is considered. In the secondary network, secondary users are considered to work in the same frequency band as the primary system. The secondary network has an ad hoc scenario with deployment of *K* antennas at each secondary transmitter. An efficient transmit beamforming technique is proposed to maximize the total throughput.

The transmit powers of secondary users are limited to a maximum value prescribed by primary users. In Figure 1(a), primary base station transmits signal to primary receivers and secondary users.  Figure 1(b) shows a transmission between secondary relays and primary receivers. Secondary users work as relays. In part (c) the transmission between secondary users is shown. The data transmission is divided into frames, whose duration is 1 time unit. *t*_*p*_ denotes the first fraction of the time slot which is dedicated to the transmission of data to both primary receiver and secondary user (SU) as the cooperative relay. Therefore the remaining 1 − *t*_*p*_ time unit of slot is separated into two subslots based on parameter *γ* as shown in Figure 2. In the second part of time slot, *γ*(1 − *t*_*p*_), relay SUs transmit primary user's data to primary receiver, and finally in the remaining part of time slot, (1 − *γ*)(1 − *t*_*p*_), multiple secondary users also access channel in space diversity mode. Here the amount of access time for secondary users is related to the contribution they made in relay process. This network coexists in the same area with secondary users which are cognitive users.

The transmission scheme is characterized by the power allocation, eigenvectors, and eigenvalues of the transmit covariance matrix.

## 3. Problem Formulation and Analysis

All secondary users are working intelligently in an ad hoc scenario. A secondary user or cognitive user is talking to a receiver using a frequency band licensed to the primary radio; the objective here is considered as to maximize the transmission capacity of the primary and secondary users and to allocate time slot for an optimal transmission. In addition, beamforming of the transmitted signal from cognitive users is considered as pre- and postbeamforming vectors The received signal at the secondary users functioning as relay is obtained as follows:

(1) $$\begin{array}{c} y_{m}\left(t_{p}\right)=\sqrt{p_{pu}}g_{pm}x_{pu}\left(t_{p}\right)+n_{m}, \end{array}$$

where *p*_*pu*_ is the transmit power of primary base station, *x*_*pu*_ and *g*_*pm*_ are the transmitted signals of the primary base station and the channel gain between primary base station and *m*th secondary user, respectively, and *n*_*m*_ denotes the additive white Gaussian noise. The received signal at the *t*′ = *γ*(1 − *t*_*p*_) time slot at primary receiver is as follows:

(2) $$\begin{array}{c} y_{p}\left(t^{\prime }\right)=\sqrt{p_{m}}g_{mp}\frac{y_{m}\left(t_{p}\right)}{\left|y_{m}\left(t_{p}\right)\right|}+n_{m}, \end{array}$$

where *g*_*mp*_ denotes the channel gain between *m*th secondary user and primary receiver, *p*_*m*_ denotes the transmit power of secondary user, and |·| represents magnitude operator. Power is constrained by a maximum transmit power limit. Here we present the pre- and postbeamforming vectors, and also we design the transmit and receive beam vectors. In fact, beam vectors associated with each secondary user are determined by optimizing a certain criterion to reach a specific target such as maximizing the throughput or minimizing the interference. To compute the beam vectors, we consider just the secondary users use MIMO system. The reason is that the interference among primary users is nulled in SINR equation. In fact, we propose an algorithm that can minimize the interference between secondary users and maximize the rate. In particular, beam vectors are selected such that they satisfy the interference free condition. Assuming that the secondary users signals are uncorrelated with zero mean, we can express the *m*th secondary user received signal at the remaining part of time slot as:

(3) $$\begin{array}{c} y_{m}^{\left(1\right)}=H_{{\text{su}}_{mm}}s_{m}+\sum_{j=1,j\neq m}^{M}H_{{\text{su}}_{jm}}s_{j}+n_{m}, \end{array}$$

where **H**_su_ ∈ *ℂ* ^*K*×*K*^, a *K* × *K* complex vector of the fading path gains between secondary users. This vector is set with random complex components and obeys the Rayleigh distribution. The additive white Gaussian noise vector **n**_*m*_ ∈ *ℂ* ^*K*×1^ is a Gaussian random process with zero mean and variance *N*_0_ on each vector component [30]. The transmit vector **s**_*m*_ of size *K* × 1 is yielded as follows:

**s**_*m*_ = *x*_*m*_ **b**_*m*_, where **b**_*m*_ ∈ *ℂ* ^*K*×1^, the prebeamforming vector, and *x*_*m*_ is the transmit sample for *m* between 1 and *M*. The *m*th receiver beam former is

(4) $$\begin{array}{c} y_{m}=a_{m}^{H}y_{m}^{\left(1\right)}. \end{array}$$

So we can express that the *m*th secondary user received signal as follows:

(5) $$\begin{array}{c} y_{m}=a_{m}^{H}H_{{\text{su}}_{mm}}b_{m}x_{m}+a_{m}^{H}\sum_{j=1,j\neq m}^{M}H_{{\text{su}}_{mj}}b_{j}x_{j}+a_{m}^{H}n_{m}, \end{array}$$

where **a**_*m*_ ∈ *ℂ* ^*K*×1^, the postbeamforming vector at the receiving secondary users. The signal to interference noise ratio (SINR) at the *m*th secondary user is as follows:

(6) Γsu=E[|amHHsummbmxm|2]E[|amH∑j=1,j≠mMHsumjbjxj|2]+E[|amHnm|2]=|amHHsummbm|2psu∑j=1,j≠mM|amHHsumjbj|2pj+||amH||2N0,

where *E*[|*n*_*j*,*m*_|^2^] = *N*_0_ and *n*_*j*,*m*_ is *j*th entry of **n**_*m*_. Also *E*[|*x*_*j*_|^2^] = *p*_su_ for *j* = 1,2, 3,…, *m*, where *E*[·] denotes statistical expectation, while (·)^*H*^ and ||·|| represent the Hermitian and 2-norm operators, respectively. So the per-user total rate is

(7) Rsu=∑m=1Mlog2(1+Γsu)=∑m=1Mlog2(1+|amHHsummbm|2psu∑j=1,j≠mM|amHHsumjbj|2pj+||amH||2N0).

The total interference plus noise covariance matrix, Φ_su_, at the *m*th secondary user is defined as follows:

(8) $$\begin{array}{c} \Phi_{\text{su}}=\sum_{j=1,j\neq m}^{M}\left(H_{{\text{su}}_{mj}}\cdot b_{j}\cdot b_{j}^{H}\cdot H_{{\text{su}}_{mj}}^{H}\right)+N_{0}I_{K}, \end{array}$$

where **I**_*K*_ is an identity matrix of size *K* × *K*. Therefore, the SINR at the *m*th secondary user can be formulated as follows:

(9) Γsu=(amHHsummbm)H(amHHsummbm)amHΦsuam=(amHHsummbm)H(amHΦsuam)−1(amHHsummbm)=bmHHsummHΦsu−1Hsummbm.

The postbeamforming vector can be expressed as follows:

(10) $$\begin{array}{c} a_{m}=\Phi_{\text{su}}^{-1}H_{{\text{su}}_{mm}}b_{m}. \end{array}$$

This gives us the following maximization of SINR at the *m*th secondary user:

(11) $$\begin{array}{c} b_{m}^{H}H_{{\text{su}}_{mm}}^{H}\Phi_{\text{su}}^{-1}H_{{\text{su}}_{mm}}b_{m}\leq \lambda_{\max}\left(j\right)b_{m}^{H}b_{m},\\ \lambda_{\max}\left(j\right)b_{m}^{H}b_{m}=\lambda_{\max}\left(j\right){||b_{m}||}^{2}. \end{array}$$

The power constraint is formulated as follows:

(12) $$\begin{array}{c} \sum_{j=1}^{M}b_{j}^{H}b_{j}=\sum_{j=1}^{M}{||b_{j}||}^{2}\leq Mp_{\max}. \end{array}$$

The maximum eigenvalue of **H**_su_*mm*__^*H*^Φ_su_^−1^ **H**_su_*mm*__ must be chosen to maximize the capacity of secondary users, and *λ*_max⁡_(*m*) is the maximum eigenvalue of **H**_su_*mm*__^*H*^Φ_su_^−1^ **H**_su_*mm*__. *P*_max⁡_ denotes the maximum transmit power of each secondary user. The transmission rate between the primary and secondary user is as follows:

(13) $$\begin{array}{c} R_{pm}=\log_{2}\left(1+\frac{p_{pu}g_{pm}^{2}}{N_{0}^{2}}\right). \end{array}$$

Similarly, the transmission rate between the secondary and primary receiver is defined as

(14) $$\begin{array}{c} R_{mp}=\log_{2}\left(1+\frac{p_{m}g_{mp}^{2}}{N_{0}^{2}}\right). \end{array}$$

The data rate of direct transmission is given by

(15) $$\begin{array}{c} R_{p}=\log_{2}\left(1+\frac{p_{pu}g_{pu}^{2}}{N_{0}^{2}}\right), \end{array}$$

where *g*_*pu*_ denotes the channel gain between primary base station and primary users. Since the transmission links are serially connected at relay, the throughput equals the smaller throughput of the two links. Hence, the overall achievable primary rate of cooperative transmission equals the minimum transmission rate of two links. Then one has:

(16) $$\begin{array}{c} R_{pu_{\text{Coop}}}\left(t\right)=\min\left\{t_{p}R_{pm},\gamma \left(1-t_{p}\right)R_{mp}\right\}. \end{array}$$

As it is clear that there is an equilibrium point between *t*_*p*_ *R*_*pm*_ and *γ*(1 − *t*_*p*_)*R*_*mp*_; therefore, *R*_*pu*_Coop__(*t*) is maximum when *t*_*p*_ satisfies *t*_*p*_ *R*_*pm*_ = *γ*(1 − *t*_*p*_)*R*_*mp*_. In order to attain maximum relayed and direct SNR, the utility functions for both the primary and secondary users with cooperative transmission rate of two transmission periods are figured out as follows:

(17) $$\begin{array}{c} U_{pu}=t_{p}\text{lo}{\text{g}}_{2}\left(1+\frac{p_{pu}g_{pu}^{2}}{n_{m}^{2}}\right)+\frac{\gamma R_{pm}R_{mp}}{R_{pm}+\gamma R_{mp}},\\ U_{\text{su}}=\left(1-\gamma \right)\left(1-t_{p}\right)\text{lo}{\text{g}}_{2}\left(1+\lambda_{\max}b_{m}^{H}b_{m}\right), \end{array}$$

where *p*_*m*_ denotes contributive transmit power of *m*th cognitive relay, *g*_*pu*_ is channel gain between primary users, and *g*_*pm*_, *g*_*mp*_ are channel gains between primary base station and *m*th secondary user and between the *m*th secondary user and primary receiver, respectively. The main cost function is defined as follows:

(18) $$\begin{array}{c} \underset{t_{p},\gamma ,b}{\mathrm{Max}}\;U_{0}\left(t_{p},\gamma ,b_{m}\right)=\alpha_{1}U_{\text{su}}+\alpha_{2}U_{pu}. \end{array}$$

In which the weights are defined as *α*_1_, *α*_2_ and *α*_1_ + *α*_2_ = 1. These parameters define the priority and importance of each utility function. So we can choose them as pairs of {(0.2,0.8), (0.5,0.5), (0.6,0.4)} arbitrarily. For beamforming, the transmitted power through all the secondary users for the *m*th secondary user is proportional to ||**b**_*m*_||^2^. The purpose of this paper is to determine optimal transmit power for all possible fading channel status in nonstationary conditions so as to maximize the channel capacity and time slot allocation with relay diversity based on particle swarm optimization. By considering a cost function, we can convert the constrained optimization process into a multiobjective cost function to meet problem constraints simultaneously. The cost function's behavior is dynamic due to nonstationary environment specifications. 

## 4. Parameters Adjustment Using PSO 

### 4.1. Brief Introduction to PSO

To achieve the optimal performance, weight multiplier *α*_*i*_ is adjusted to satisfy priority. A swarm of particles that represent potential solutions are evolved in the search space. Particle swarm optimization is inspired by observing the bird flocking or fish school. Scientists found that the synchrony of flocking behavior was through maintaining optimal distances between individual members and their neighbors. Furthermore, they simulated the scenario in which birds, fishes, or bees search for food, and observing their social behavior, they perceived that in order to find food, the individual members determined their velocities by two agents, their own best previous experience and the best experience of all other neighbors and members. According to this concept, the so-called PSO for optimization of continuous nonlinear functions has been developed [31, 32]. However, several improvements have been made in this work, we used the original one, and employing the improved versions of PSO for this optimization remained, an open problem for future studies [33]. Here birds, fishes, or bees are called particles, each representing a potential solution, and they all have their position, velocity, and fitness value. To find the optimal solution, each particle adjusts its flying according to its own flying experience and companion's flying experience. A swarm of particles are created. While the initial particles are randomly generated in the search space, each particle keeps its best position in its memory. We should check to ensure that all the particles stay inside the search space, if a particle is outside the search space. It is moved back inside the search space randomly and finally it is forced to stay at the border. In Algorithm 1, the procedure is defined. Based on its searching mechanism, primitive position denotes solution and velocity denotes the mutative direction the solution may take. The pseudocode of the PSO procedure can be written as follows.

In a *d*-tuple searching space the position and velocity of a particle *i* are denoted by *X*_*i*_ = [*x*_*i*1_, *x*_*i*2_,…, *x*_*id*_] and *V*_*i*_ = [*v*_*i*1_, *v*_*i*2_,…, *v*_*id*_]. Our fitness function is to evaluate every particle to figure out the best solution *P*_*i*_ = [*p*_*i*1_, *p*_*i*2_,…, *p*_*id*_], it may find the best solution *P*_*g*_ for the whole swarm at time *t*, and the position and velocity are updated with (19) and (20).*P*_*g*_ is the optimum solution. Consider

(19) vij(t+1)=ωvij(t)+c1r1[pij−xij(t)]+c2r2[pgj−xij(t)],

(20) $$\begin{array}{c} x_{ij}\left(t+1\right)=x_{ij}\left(t\right)+v_{ij}\left(t+1\right),\;j=1,\ldots ,d, \end{array}$$

where *ω* denotes the inertia weight factor, *c*_1_, *c*_2_ denote positive accelerators, and *r*_1_, *r*_2_ are random numbers uniformly distributed in interval [0,1]. The role of inertia weight *ω* is considered to be crucial for convergence and is to control the impact of the previous history of velocity on the current velocity. Thus it regulates the tradeoff between global and local exploration for the swarm. A large *ω* makes the searching escape from local minima and facilitates global searching, while small *ω* facilitates local searching and convergence. When the particles get trapped in local optima, the inertia weight is augmented, and when they are dispersive, the weight is decreased. The velocity interval [*v*_min⁡_, *v*_max⁡_] and position interval [*x*_min⁡_, *x*_max⁡_] are to restrict the searching in the required domain. The above velocity renewal equation (19) comprises three parts. The first part is dominated by current velocity and contributes to the tradeoff between global searching and local searching while the second part embodies cognitive pattern and is to adjust direction based on its recollection to avoid local minima. The third part reflects social effects that shared information contributes to collaboration.

### 4.2. Allocation Algorithm

According to discussion in Sections 3 and 4, in this simulation for the adaptation of parameters to the PSO, some parameters are selected as particles, so we adapt these parameters to PSO particles and try to update them as iteration goes on. In each iteration of the algorithm, we make the answer after calculating the average amount of particles for specific number of repeating. Our proposed particle is a vector which contains the power of secondary users *p*_*m*_, the prebeamforming vector **b**_*m*_, the time slot division factor *γ*, and the time slot duration *t*_*p*_. The prebeamforming vector **b**_*m*_ is set with random complex components by PSO which is one part of the particle. Each component of particle [*p*_*m*_, **b**_*m*_, *γ*, *t*_*p*_] has a velocity denoted by *v*_*ij*_(*t*) and position denoted by *x*_*ij*_(*t*). Each particle remembers its best position visited so far, denoted by *p*_*ij*_, which provides the cognitive information. Every particle also knows the best position visited so far among the entire swarm, denoted by *p*_*g*_, which provides the social information. *p*_*ij*_, *p*_*g*_ are updated at each iteration. Also each element of particle is checked to ensure that it stays inside the search space. The amount of *p*_*m*_ ≤ 5 mW, 0 ≤ *γ* ≤ 1, and 0 ≤ *t*_*p*_ ≤ 1 and complex component of vector **b**_*m*_ should be considered for boundary check. For each term of particle the boundary region is defined. As it is clear, selected particle consists of a scalar and vector part. Using (19) and (20), the adaptation parameters to position and velocity interval are set [30, 34, 35]. Because our simulation is run in a nonstationary environment, all channel fading gains are generated randomly with Rayleigh distribution. So during the simulation, the amount of these gains may be changed, and as a result the algorithm will track the cost or fitness function, as all mentioned parameters vary. 

## 5. Numerical Results

**Figure 3:**
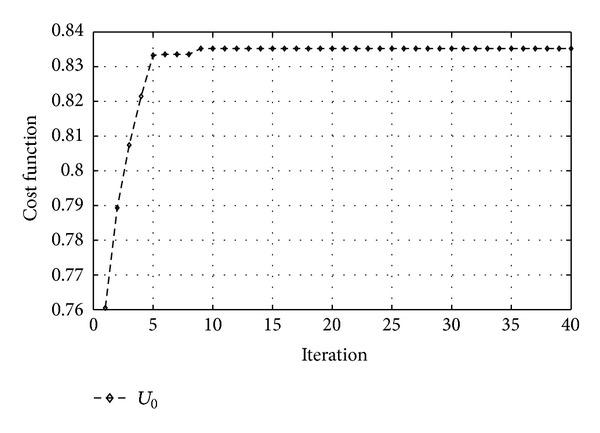
Behavior of the main cost function.

In a simulation model, we consider some simulation results to investigate performance of the proposed scenario in which the primary and secondary users are randomly distributed over the considered area. A secondary network coexists and/or shares the radio spectrum with a primary network to which the spectrum is licensed, in an infrastructure scenario. The channels between the transmitters and receivers are assumed to be Rayleigh faded with mean of one. The channel gains are independent across subchannels. The power spectral density of additive Gaussian noise is 10^−8^ W/Hz. Empirically, our results suggest that the velocity limit can be set to *v*_*m*_ = 0.09 and the acceleration coefficients can be set to *c*_1_ = 2 and *c*_2_ = 2. The primitive inertia weight *ω* is set to 0.9, ranges from 0.3 to 0.9, and varies as the iteration goes on. Path noises are independent zero-mean complex Gaussian random variables with variance *N*_0_ = 1. The maximum transmit power *P*_max⁡_ for each secondary user is assumed to be 5 mW, and the transmit power of primary base station is set to 0.2 W for a 10^5^ Hz bandwidth. Interference from primary users to base station is ignored. The amounts of time slot division parameters, *γ* and *t*_*p*_, will be set during the iterations by related components in selected particle. It was found that the more repetition of the algorithm in each iteration has the much accuracy. In our algorithm, we repeated each iteration to reach the best accuracy. In Figure 3 the behavior of the cost function is shown. 

**Figure 4:**
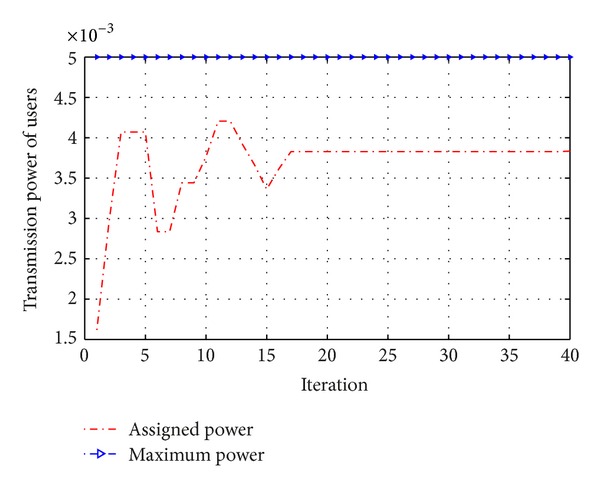
Convergence of transmission power for secondary users.

By making average global information of the best particles, the accuracy of the best particles has been raised as shown in Figure 4. We can see that the behavior of the cost function and its convergence are the attributes of PSO, and it is clear that the all constraints are fulfilled.

**Figure 5:**
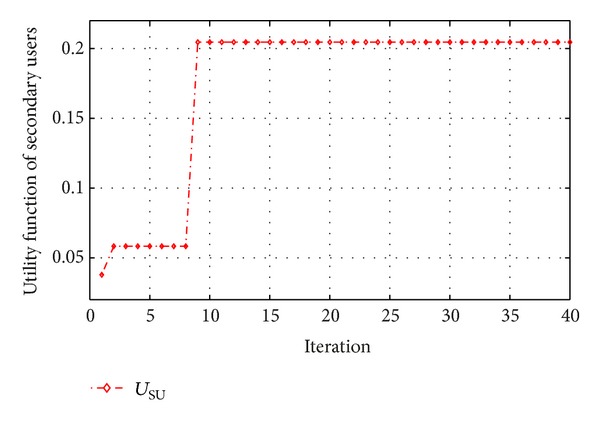
Utility function of the secondary users.

Figure 4 shows that there is a difference between maximum transmission power of users and assigned transmission power. This convergence shows the control of the transmission power for secondary users by the proposed algorithm. From Figure 5, it can be seen that the utility function of secondary users arises by increasing the amount of transmission power; however, the power will be limited by our simulations constraints. 

**Figure 6:**
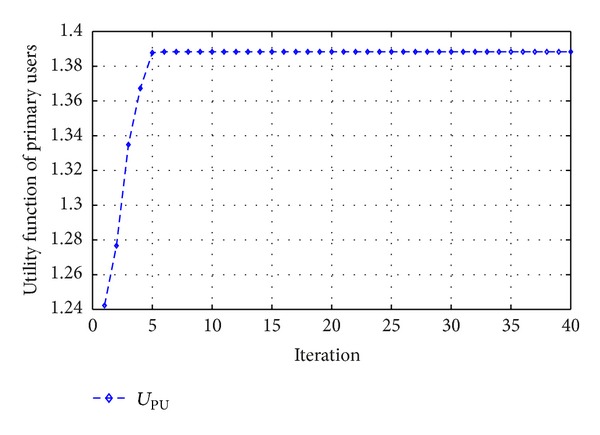
Utility function of the primary users with cooperative relays.

**Figure 7:**
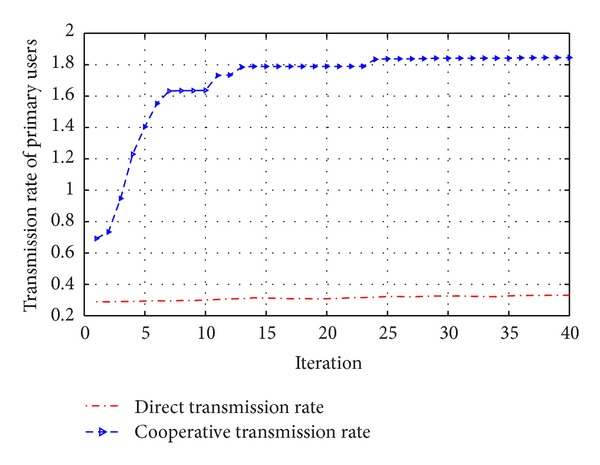
Transmission rate of primary users.

From Figure 6, it can be seen that the utility function of primary users arises along with cooperative relays. Furthermore, the fitness functions steer the evolution of the PSO in the correct direction to optimize the given multiobjective cost functions for the secondary and primary users with the defined constraints in a nonstationary environment.  Figure 7 shows the performance of cooperative relay. It can be seen that the utility function of primary user arises when cooperative relay is used. However the channel gains are Rayleigh faded in a nonstationary environment. In wireless communication systems, the wireless channel varies quickly, so cognitive radio network requires exhausting operation every few seconds, Also there is a tradeoff between computational complexity and power consumed in this wireless communication scenario. Therefore it might be considered as the next interest. 

In our simulation for short time slots, we consider slow varying channels and the algorithm can set the transmission parameters.

## 6. Conclusions 

 We have proposed a PSO-assisted distributed minimum transmission power and time slot allocation with relay diversity in cognitive or secondary radio network in a randomly time varying environment. The scenario is formulated in the downlink mode of the primary user network to maximize the transmission capacity of secondary and primary users. However, the minimum transmission power of each cognitive or secondary user is considered. We have developed joint time slot and transmission power control with relay diversity in which PSO adjusts the parameters, while maintaining a quality of service for the primary user. The PSO-aided algorithm provides improved performance by using appropriate pre- and postbeamforming. The proposed scheme shows the performance of a heuristic improvement in cognitive radio performance in a dynamic environment. During an extended searching space, the fitness function determines the values, and the number of iteration determines the speed. Hence, the idea of considering the practicality of the proposed method in terms of computational complexity is one of the next interests to us.

## Figures and Tables

**Algorithm 1:**
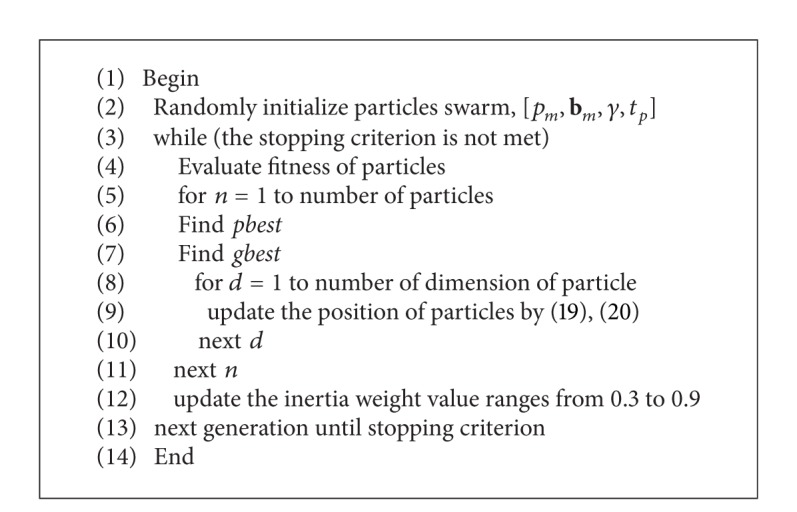
Pseudocode for PSO.
